# Formulation Approaches to Crystalline Status Modification for Carotenoids: Impacts on Dissolution, Stability, Bioavailability, and Bioactivities

**DOI:** 10.3390/pharmaceutics15020485

**Published:** 2023-02-01

**Authors:** Wan-Yi Liu, Yun-Shan Hsieh, Horng-Huey Ko, Yu-Tse Wu

**Affiliations:** 1 School of Pharmacy, College of Pharmacy, Kaohsiung Medical University, Kaohsiung 80708, Taiwan; 2 Department of Fragrance and Cosmetic Science, College of Pharmacy, Kaohsiung Medical University, Kaohsiung 80708, Taiwan; 3 Department of Medical Research, Kaohsiung Medical University Hospital, Kaohsiung Medical University, Kaohsiung 80708, Taiwan; 4 Drug Development and Value Creation Center, Kaohsiung Medical University, Kaohsiung 80708, Taiwan; 5 Center for Cancer Research, Kaohsiung Medical University, Kaohsiung 80708, Taiwan

**Keywords:** carotenoids, crystallization status modification, formulation approaches

## Abstract

Carotenoids, including carotenes and xanthophylls, have been identified as bioactive ingredients in foods and are considered to possess health-promoting effects. From a biopharmaceutical perspective, several physicochemical characteristics, such as scanty water solubility, restricted dissolution, and susceptibility to oxidation may influence their oral bioavailability and eventually, their effectiveness. In this review, we have summarized various formulation approaches that deal with the modification of crystalline status for carotenoids, which may improve their physicochemical properties, oral absorption, and biological effects. The mechanisms involving crystalline alteration and the typical methods for examining crystalline states in the pharmaceutical field have been included, and representative formulation approaches are introduced to unriddle the mechanisms and effects more clearly.

## 1. Introduction

**Figure 1:**
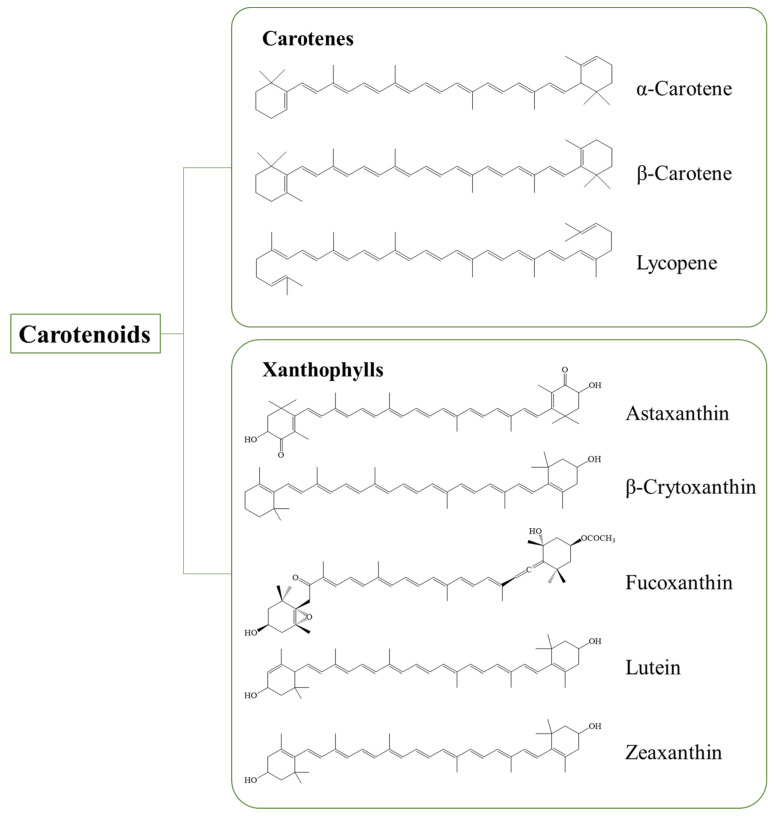
The structure of carotenes and xanthophylls.

Carotenoids are composed of compounds containing typically 40 carbon atoms and are synthesized by plants or microorganisms. The structure of carotenoids commonly includes a central carbon chain with several conjugated double bonds and they partially have different cyclic or acyclic end groups [1]. Carotenoids are divided into carotenes and xanthophylls depending on the chemical structure as presented in Figure 1. Carotenes, such as α-carotene, β-carotene, and lycopene, only contain carbon and hydrogen in their structure without oxygen atoms. Xanthophylls, such as astaxanthin, β-cryptoxanthin, lutein, and zeaxanthin, are the other type; they are carotenoids containing one or more oxygen atoms in their structure. The bioactivities of carotenoids have been demonstrated to be associated with chemical structures, such as the number of conjugated double bonds and the types of functional groups at the ends [2].

Carotenoids exhibit well-known anti-oxidative activities and are most likely involved in scavenging the singlet oxygen and peroxy radicals [3]. Reactive oxygen species (ROS) are generated during normal metabolism and engaged in enzymatic reactions, mitochondrial electron transport and signal transduction. Excessive ROS would damage biologically essential factors and elevate the risk of degenerative diseases [4]. Therefore, carotenoids are considered excellent antioxidants that benefit various diseases associated with oxidative stress. The bioactivities of carotenoids have been reported in previous studies and are listed in Table 1.

Carotenoids commonly exist in crystalline states and exhibit an ordered intermolecular arrangement in the solid state. Various intermolecular interactions, such as hydrogen bonding and π-π stacking interactions, maintain the structure integrity and crystalline status of carotenoids [5,6]. The crystalline status of carotenoids contributes to the scant solubility and dissolution, which further restricts their oral bioavailability and health-promoting effects. Therefore, improving the solubility of carotenoids is a critical issue for achieving the desired plasma concentration in systemic circulation to attain the expected biological effects [7]. The higher lattice energy of crystalline compounds usually correlates with their poor solubilities, because the energy has to be overcome before the compound can be dissolved in the medium [8]. Carotenoids have poor aqueous solubility owing to the higher lattice energy. Thus, the crystalline status is a crucial factor for the application of carotenoids.

**Table 1:** The bioactivities of carotenoids that have been reported in previous studies.

Carotenoids	Dose	Model	Bioactivities	Reference
Astaxanthin	1.0 mg/mouse/day	Diabetic C57BL/KsJ-db/db mice	Anti-diabetic (Blood glucose↓ and preservation of -cell function)	[9]
Astaxanthin	2 mg	Healthy women	Immune response improvement (Mitogen-induced lymphoproliferation↑ Natural killer cell, total T and B cell↑ DNA damage biomarker↓)	[10]
Astaxanthin	5 μM	Primary hippocampal neurons	Treatment of Hcy-mediated neurological disorders (ROS and superoxide anion↓)	[11]
β-Carotene	45 mg/day	Healthy older adults	Immunostimulant (Total T cells and NK cell↑)	[12]
β-Carotene	200 mg/Kg	Male albino mice	Anticonvulsant activity (Duration of general tonic–clonic seizures↓ General tonic–clonic seizures latency↑)	[13]
β-Carotene	30 μM	Human prostate cancer cell line (PC-3 cell)	Anticancer (cell viability: 51.4%)	[14]
β-Carotene	2.05 mg/Kg	Male albino mice	Treatment of Alzheimer’s disease (Acetylcholinesterase and amyloid β-protein↓)	[15]
β-Cryptoxanthin	0.8 mg/Kg/day	Male mice	Anti-obesity (Adipocyte hypertrophy↓)	[16]
Fucoxanthin	5 μM	Human fibroblast	Protection against UVB radiation-induced oxidative stress (ROS↓)	[17]
Fucoxanthin	1.06-2.22%	C57BL/6J mice	Anti-obesity and anti-diabetic effects (Body weight and white adipose tissue↓ MCP-1 expression↓ and Adrb3 and GLUT4↑)	[18]
Fucoxanthin	0.2%	C57BL/6N mice	Anti-obesity (Fatty acid β-oxidation activity and lipogenic enzyme activities ↓)	[19]
Lutein and Zeaxanthin	Oral: lutein 100 ppm zeaxanthin 6 ppm Topical: lutein 10 ppm zeaxanthin 0.6 ppm	Healthy women	Photoprotective (Lipid peroxidation↓ skin lipid, skin hydration and skin elasticity↑)	[20]
Lutein and Zeaxanthin	Lutein: 5% zeaxanthin: 0.2%	β5−/− mice	Prevention of age-related retinal pigment epithelium actin damage (4-hydroxynonenal-adduct formation, age-related cone and rod photoreceptor dysfunction ↓)	[21]
Lutein and Zeaxanthin	Lutein:10 mg Zeaxanthin: 2 mg	Healthy older adults	Improvement of cognitive function (Macular pigment optical density, complex attention and cognitive flexibility domains↑)	[22]
Lycopene	5 μg/mL	Fungal cell (*Candida albicans*)	Antifungi (Destruction of fungi membrane and inhibition of the normal budding process)	[23]
Lycopene	2 μM	Rat cortical neurons	Treatment of Alzheimer’s disease (Intracellular ROS and superoxide production↓)	[24]
Lycopene	0.2 or 0.5 μM	Neuronal SH-SY5Y cells	Neuron protection (ROS↓ and mitochondrial dysfunction ↓)	[25]
Lycopene	0.03% (*w/w*, mixed into normal chow)	Male C57BL/6J mice	Treatment of Alzheimer’s disease (Memory loss behavior, amyloid plaques, amyloid precursor protein, neuronal β-secretase BACE1, inflammatory mediators and oxidative stress↓, α-secretase ADAM10↑)	[26]
Lycopene	2 μM	Mice cerebral cortical neurons	Neuron protection (Nerve growth factor, brain-derived neurotrophic factor, and vascular endothelial growth factor excretion↑ and anti-apoptosis)	[27]
Lycopene	100 mg/Kg	Female Sprague-Dawley rats	Treatment of vascular dementia (Oxidative stress in hippocampus↓)	[28]

↑: enhancement. ↓: reduction. ROS: reactive oxygen species. Adrb3: β3-adrenergic receptor. GLUT4: glucose transporter 4.

## 2. Effects of Crystalline Status Modification on the Physicochemical Properties of Carotenoids

**Figure 2:**
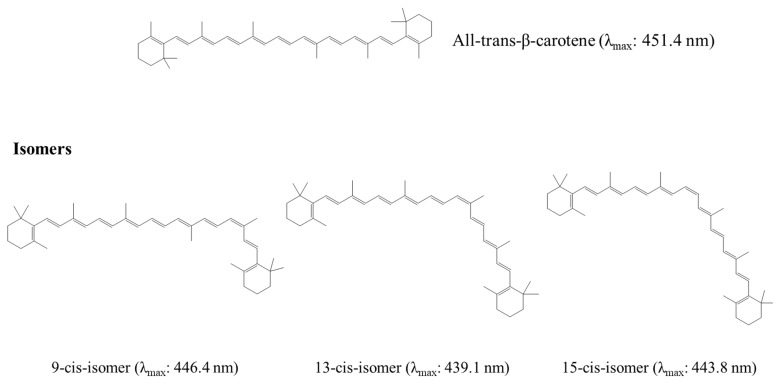
The structures of E (*trans*)- and Z (*cis*)- forms of β-carotene.

The chemical structures of carotenoids possess many chiral centers, which result in a variety of conformations. The *cis*-form (Z-form) and *trans*-form (E-form) have been demonstrated to affect the crystalline state and further have an impact on the physicochemical properties. In general, the all-*trans* carotenoid isomers are the most stable ones owing to their different Gibbs free energies and exist commonly in nature. Only 5-*cis*-lycopene was found to be more stable than all-*trans*-isomers [29]. The *cis*-form isomers display diverse properties compared to the *trans*-form isomers, such as a shallower color caused by the shorter maximum absorption wavelength and smaller extinction coefficient [30], reduction in the crystalline ratio, lower melting point, and poor stability [31]. Taking β-carotene as an example, Figure 2 shows the isomers of β-carotene and the maximum absorption wavelength of these compounds [32]. A previous study reported the transformation method and different properties of the E-form and Z-form carotenoids. The Z-form lycopene has been discovered to have a 4000-fold higher solubility compared to the E-form in ethanol [6]. The lower degree of crystallinity leads to higher solubility in bile acid micelles, and the higher solubility would further result in greater bioaccessibility. Interestingly, the ideal bioaccessibility cannot completely correlate with bioavailability [6]. The cellular uptake efficiency is a critical factor that influences bioavailability, and the efficiency depends on the molecular structure and hydrophobic properties. In previous studies, Yang et al. [33,34] found that the *trans*-lutein had better passive diffusion into enterocytes due to the linear structure, and the affinity to transporters in the intestine changed the cellular uptake efficiency as well because the relatively higher solubility of 9Z-astaxanthin caused poorer affinity to the transporter compared with 13Z-astaxanthin and the *trans*-isomer. This theory can also be applied to β-carotene. E-β-carotene was also reported to exhibit higher absorption than the Z-form in both in vitro [35] and in vivo studies [36]. Therefore, modification of the crystalline conformation impacts the solubility, dissolution, intestinal absorption, and further bioavailability of carotenoids, together with the bioactivity.

**Figure 3:**
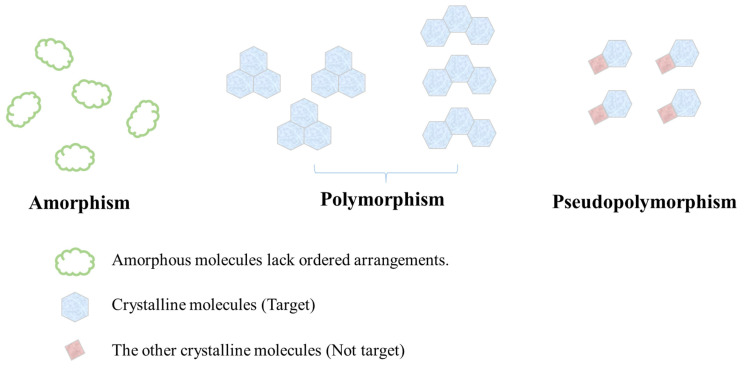
The difference among amorphism, polymorphism, and pseudo-polymorphism.

Micronized crystalline lutein has been prepared to improve its dissolution and oral bioavailability [37]. A wet-jet milling method with high mechanical force was applied to reduce the particle size, and the procedures also converted the crystalline form into the polymorphic state, which belonged to a metastable situation with higher energy. The effects of size reduction and crystalline transformation are beneficial to dissolution and oral absorption. Though the solubility may have effects on the transporter affinity, the absorption still requires the transition from solid form to solution [38]. In order to improve the aqueous solubility of active crystalline compounds, the alternation of the crystalline condition is the simplest method. Crystalline states can be classified into three types—polymorphic state, pseudo-polymorphic state, and amorphous state—the difference among the three states is shown in Figure 3. Polymorphs are the same constituents with different crystalline arrangements, and they often benefit solubility. Notably, the improvement is small due to the small energy difference between the polymorphs [39]. Pseudo-polymorphs include hydrates or solvates, and they are usually unwanted crystalline forms. In the case of solvates, this depends on the characteristics of solvents. On the other hand, water forms hydrogen bonds with the active compounds in hydrates, and the lattice enthalpy would escalate to decrease the solubility, accompanied by a lower bioavailability [5]. The amorphous state lacks the ordered arrangement of molecules, causing amorphs to have poor thermodynamic stability, and it is usually the first choice to greatly enhance the aqueous solubility [40]. The amorphous state is divided into molecularly pure, which only transfers the crystalline state of the active compounds and formulation. Both of them may increase the solubility; however, the stability and scale-up of the molecularly pure type are hard to achieve [41]. Therefore, preparing a formulation to transfer the crystal to another state is quite an important technique to enhance the solubility and oral absorption, and then exhibit the expected bioactivities. To distinguish the crystalline status in the formulation, detection methods have been introduced.

## 3. Methods for Examining the Crystalline Status

Powder X-ray diffraction (PXRD), differential scanning calorimetry (DSC), thermogravimetric analysis (TGA), transmission electron microscopy (TEM), and scanning electron microscopy (SEM) are fundamental methods to determine the characterization of the crystal in pharmaceutical formulations; usually, more than two methods are adopted to reveal more reliable results.

### 3.1. PXRD

PXRD is a well-known method to qualify the crystalline state and quantify the crystallinity as well as crystal size in a formulation. Fingerprint data of APIs can be obtained by PXRD, and possible polymorphs can be identified. The intensities of diffraction peaks are positively related to crystallinity, and we can use them to calculate the percentage of amorphization. However, nanocrystals and amorphism are hard to differentiate because the low crystallinity leads to broad peaks (i.e., Scherrer broadening) [42]. The nanocrystal size can be calculated using the Scherrer equation (Equation (1)), and the formula is only viable for nanocrystals around 100–200 nm [43].

τ = kλ/(β cosθ)(1)

where τ is the average size of the crystal; k is the shape factor; λ is the wavelength of the X-ray; β is the line broadening at half the maximum intensity in radians; θ is the Bragg angle.

**Figure 4:**
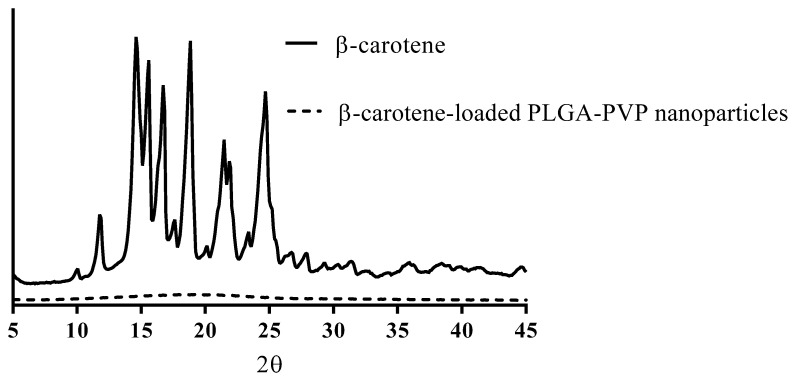
PXRD profiles of β-carotene and its nanoformulations.

Figure 4 shows the PXRD profiles of β-carotene and nanoformulations, where the API group presents sharp and strong peaks compared with the nanoformulation groups, indicating the API has been encapsulated in the formulations in an amorphous state.

### 3.2. Electron Microscopy—TEM and SEM

For nanoscale crystals, electron microscopy is applied to visualize the lattice. The crystal size and the structure of the lattice are provided by TEM. The sizes calculated using TEM may be larger than those from the Scherrer equation as particles seen in the TEM images are possibly not crystals, and the crystalline imperfections broaden the peaks in PXRD, causing calculation bias [44]. SEM is a technology suitable for morphology observation, and the relationship between phases such as erosion can be further acquired compared with TEM [45]. However, the two technologies are limited by the samples “seen” by the microscope, and only unilateral information may be obtained. Thus, more significant sample sizes and observed angles are needed to avoid sampling errors and to obtain the whole morphology.

### 3.3. Thermal Methods—DSC and TGA

**Figure 5:**
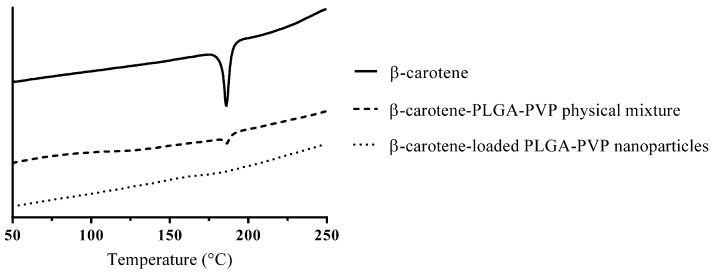
DSC thermograms of β-carotene, a β-carotene-PLGA-PVP physical mixture, and β-carotene-loaded PLGA-PVP nanoparticles.

Thermal methods detect the thermal behavior of the whole sample, and they will not encounter issues with the difference between the surface and the core or the sampling bias [46]. DSC measures the heat required for the temperature increase in the samples; the heat absorption from melt formation or release due to the crystallization will be detected [42], and T_m_ (melting point), T_g_ (glass transition temperature), T_c_ (crystallization temperature), and T_d_ (degradation temperature) can be obtained [47]. The endothermic T_m_ peak disappears once the crystals convert into amorphs, and the concept can be applied to confirm the encapsulation of amorphous APIs in formulations [48]. Taking DSC thermograms (Figure 5) as an example, the peak standing for the melting point of β-carotene at 186 °C was observed in the crystalline API and physical mixture, and the peak disappeared in the formulation group. However, it is hard to distinguish the amorph merely from the thermogram when the degree of crystallization is relatively high, and a simple formula (Equation (2)) can be used to calculate the amorphous content [49].

(2) $$\mathrm{Amorphous}\;\mathrm{content}=\frac{\Delta H_{f}^{\mathrm{amorphous}}}{\Delta H_{f}^{\mathrm{crystal}}}$$

where $\Delta H_{f}^{\mathrm{amorphous}}$ represents the enthalpy of the fusion of the amorphous fraction, and $\Delta H_{f}^{\mathrm{crystal}}$ represents the enthalpy of the fusion of the crystalline fraction.

TGA measures the weight loss resulting from heating, and this technology can be used to investigate crystals containing volatile substances [5]. It is also often conducted combined with DSC to further confirm the thermal behavior. The crystal and amorph can be distinguished by the different weight loss under the same temperature [50], which can be used to prove the complex formation [51].

## 4. Effects of Preparation Factors on Crystalline Status of Active Pharmaceutical Ingredients

**Figure 6:**
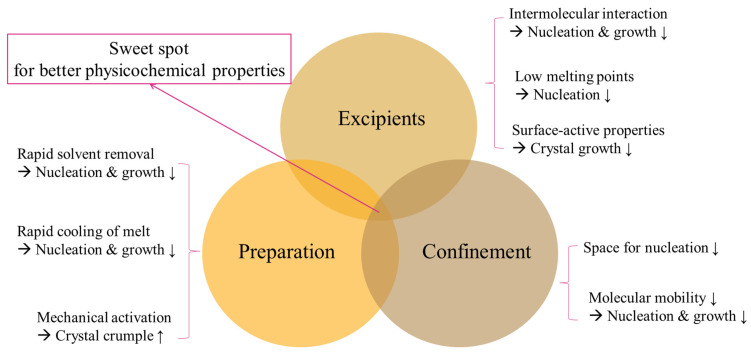
Formulation conditions affecting crystallization.

Turning active pharmaceutical ingredients (APIs) into amorphous or polymorph states is expected to change their biopharmaceutical properties, including their dissolution rate and bioavailability, which can be accomplished using various manufacturing processes. Major preparation factors that affect the crystalline status are summarized in Figure 6.

### 4.1. Excipients

Crystallization often includes two major steps, namely, nucleation and growth; the addition of excipients can manipulate the crystallization of APIs. The rate of nucleation is usually positively related to molecular mobility, suggesting that the restriction of molecular mobility through intermolecular interaction between excipients and APIs can also affect the crystallization of APIs.

Poly(ethylene oxide) as a plasticizer increases the nucleation rate due to its water-absorbing properties [52]. Amphiphilic polymers are preferred for hydrophobic carotenoids because the hydrophobic substituents of the excipients increase the interaction with carotenoids, and the hydrophilic substituents interact with water to enhance the dissolution [53]. In addition, lipids act as inhibitors or promoters of crystallization. The choice of lipids is based on three principles: hydrocarbon chain lengths, unsaturated degrees, and esterification degrees [54]. Saturated fatty acids with high polarity and a short chain length (e.g., butyric acid) have lower melting points, and the lower melting point prevents the molecule from forming crystalline nuclei. Meanwhile, fatty acids can adsorb on the interface of the liquid—solid nucleus to inhibit crystalline growth because of their surface-active properties [55].

Moisture also plays an essential role in crystalline formation. When water and APIs form crystals together, hydrates are produced, and the hydrogen bonding between water and APIs leads to higher lattice enthalpy and poorer bioavailability [5]. Hydroxyl groups of hydroxypropyl methylcellulose (HPMC) inhibit hydrate formation by occupying the sites where APIs and water have hydrogen bindings. The higher molecular weight of the excipients provides more functional groups for interactions and the full surface coverage of APIs [56]. Excipients with charges, such as dextran, alginate, and chitosan, can form ionic interactions with APIs possessing opposite charges, therefore, preventing the ongoing crystallization of APIs [56]. As excipients have great impacts on the stabilization of amorph conditions, excipient screening is an essential step for developing optimal formulations.

### 4.2. Preparation

Dissolution followed by rapid precipitation, melting followed by rapid cooling, and direct solid conversion are major methods to change the crystalline status of APIs. For the technique of dissolution followed by rapid precipitation, excipients dissolved in organic solvents loosen their structure, having interactions with the solvents. Meanwhile, APIs dissolved in organic solvents (i.e., amorphous states of APIs) enter the structure of the excipients and integrate into loosened excipients. With the rapid removal of solvents, the excipient and the amorphous APIs do not have enough time for ordinary crystallization and finally form a compact structure [57].

For the method of melting followed by rapid cooling, excipients and APIs are melted via heating. In the melt phase, the heat loosens the structure of the excipients, allowing APIs to occupy the space inside the excipients. Thereafter, fast cooling of the melt dramatically increases the viscosity and decreases the volume in a short time, resulting in much slower molecular mobility and molecule arrangement to prevent nucleation and crystalline growth [58].

Direct solid conversion refers to mechanical activation by milling. During the milling process, local heat accompanied by cooling results in amorphization, and the procedure increases static disorder and intrinsic dynamic disorder to the threshold value of the lattice, leading to the crumpling of the crystals. A limitation of this method, which should be taken into consideration, is the possibility of incomplete crystalline disorder [59]. The milling processing only renders the surface of the ingredients amorphized and may lead to inconsistent results in the physicochemical characterization [46].

In fact, for APIs, different manufacturing processes will produce varying degrees of amorphous forms, which cause diverse profiles in solubility, dissolution, and bioavailability [60].

### 4.3. Confinement (Change in Particle Sizes)

**Figure 7:**
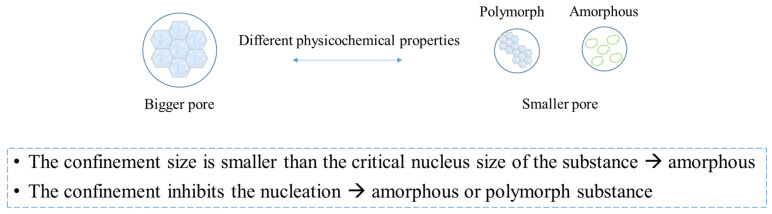
The concept of confinement and the relationship between confinement and crystals.

Confinement in the pharmaceutical field represents the physical restriction of APIs at the indicated scale. Under confinement, a different polymorph or amorph may appear, and confinement represents a practical handle to control or stabilize crystalline growth, as shown in Figure 7. The reasons why confinement affects the crystalline behavior remain unclear, but there are some proposed mechanisms: (i) When the size of the confinement is smaller than the critical nucleus size of the most stable crystals, the crystalline growth will be inhibited and a new polymorph or amorph will possibly form [61]. However, all polymorph forms of acetaminophen can grow under nanoconfinement [62]. The opposite result may relate to the chosen size for the confinement. Several pore sizes have been studied for the crystalline behavior of nifedipine, and McKenna and his colleague found a new polymorph presented at a certain pore size [63]. (ii) APIs in each compartment have to nucleate independently. As long as the compartment walls are not nucleated, homogeneous nucleation will dominate, which takes longer for crystallization. (iii) A thickness of 1 nm immobilizes the surface layer with high surface energy possibly forming at the compartment walls, and the immobilized layer slows the crystallization kinetics significantly [63,64]. The nanocompartments of the liposome truly inhibit crystalline growth [65]. Praziquantel occurs in its amorphous form under nanoconfinement because of its larger crystalline lattice, and amorphous praziquantel was found to increase the dissolution rate by five-fold [66]. This strategy can also be applied to hydrophobic carotenoids. Next, the application of these theories in carotenoid formulations will be introduced based on different types of formulations.

## 5. Approaches for the Crystalline Status Modification of Carotenoids

### 5.1. Co-Crystallization

Co-crystallization is defined as a single structurally homogeneous crystallization containing at least two neutral units (API and excipients) existing in solid and definite stoichiometric amounts [67]. This method is usually accomplished via supersaturation, which refers to slow cooling until the solubility limit is reached. Usually, the solubility of products prepared using this approach may not be significantly increased owing to the existence of a crystalline lattice structure, but it may provide several advantages, such as ease of preparation, lower hygroscopicity, and greater chemical stability of the products [68]. The commonly used excipients for co-crystallization systems should contain specific functional groups, including carboxylic acids (e.g., acetic acid and salicylic acid), amides (e.g., nicotinamide, saccharin, and urea), and alcohols (e.g., mannitol and sorbitol) to form intermolecular bonds with APIs [69].

The utilization of a supersaturated sucrose solution has been proposed for the preparation of carotenoid-rich extracts via the co-crystallization method [70], which aims to improve the dispersibility, hygroscopicity, and thermal stability of β-carotene. The ordered crystal of sucrose is transformed into an irregular and porous structure after the incorporation of β-carotene during the cooling and recrystallization processes. The crystalline status of co-crystallization can be evidenced by DSC and XRD examination. Though the technique of preparing pharmaceutical co-crystals with sucrose is believed to improve the solubility, dissolution, and other physicochemical properties of the encapsulated materials [71], merely the dissolution kinetics of sucrose have been determined in the current literature. One possible reason might be that the true solubility of the cocrystal products is not readily determined because API tends to transform into the most stable form in solution [72]. Few studies on carotenoid-loaded co-crystallization have discussed the crystalline state. Therefore, it may be an unexplored frontier and require more investigation for further discussion.

### 5.2. Solid Dispersion

**Figure 8:**
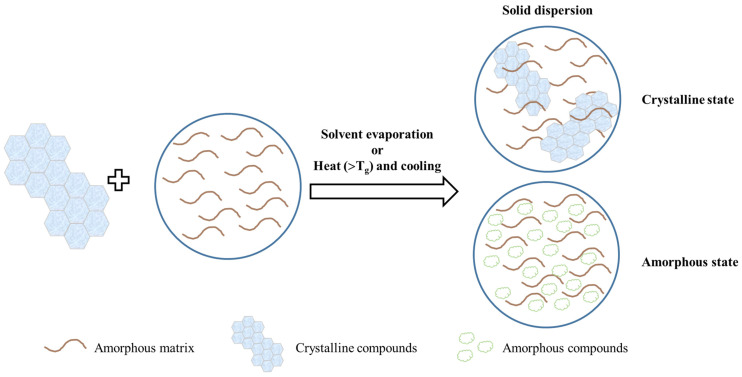
The scheme of solid dispersions.

Solid dispersion is a commonly used technology for crystalline state alternation and is defined as the dispersion of APIs in an inert carrier, such as sugars, polymers, and surfactants (Figure 8). Solvent evaporation and hot-melt methods are commonly operated, and the amorphous state may be produced during solvent removal or cooling [41]. The interaction between polymers and APIs generally results from the occurrence of hydrogen bonds and hydrophobic interaction. When solid dispersions are placed into an aqueous medium, such as simulated gastric fluid or simulated intestinal fluid, they would rapidly dissolve and exist in the supersaturation state owing to the amorphous state occurrence. Therefore, this may increase the aqueous solubility of poorly soluble APIs. Some polymers have been reported to retard crystalline growth in several ways: polyvinylpyrrolidone (PVP) suppresses the nucleation process and HPMC adsorbs on the surface of the crystal to prevent the formation of crystals [73]. In addition, the nucleation and growth procedures may be retarded via hydrogen bonding between APIs and excipients, which further inhibits crystalline formation [74].

It has been reported that β-carotene containing a solid dispersion composed of PVP and sucrose fatty acid ester (S-1670) was prepared by hot-melt extrusion and was found to be in an amorphous state via DSC and XRD. It was also proved to enhance solubility by about 390-fold, dissolution behavior, and also oral bioavailability [75,76,77]. A solid dispersion prepared using cyclic amylopectin has been used to protect β-carotene from light, heat and oxidation. The crystalline state of the solid dispersion was hard to detect using XRD owing to the uniform distribution in cyclic amylopectin. Starch was reported to inhibit the crystallization of a water-insoluble compound, β-carotene, and the composite was formed in an amorphous state. Cyclic amylopectin with a hydrophobic internal core could bind with β-carotene and hydrophobic compounds via intermolecular forces to generate a more amorphous formation [78].

Chang et al. [79] prepared lycopene dripping pills consisting of PEG 6000, Cremophor EL, and Tween 80 to improve the release behavior and oral bioavailability by approximately six-fold. The dripping pills were determined to be in an amorphous form via SEM and DSC. In this study, it was demonstrated that the lower viscosity caused by excessive emulsifiers may facilitate recrystallization.

### 5.3. Inclusion Complex

**Figure 9:**
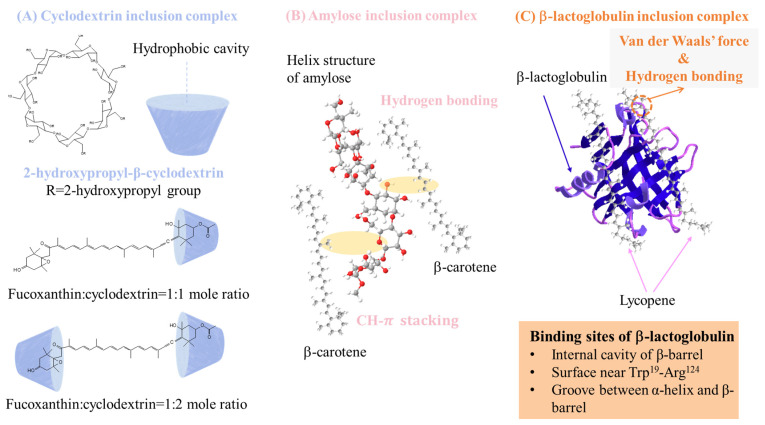
Scheme diagrams of (**A**) cyclodextrin, (**B**) amylose, and (**C**) β-lactoglobulin inclusion complexes [81,82,83].

A complex is defined as the combination of APIs and ligands through hydrogen bonding, van der Waals forces, or hydrophobic effects [80]. Only a few compounds can be used as ligands to encapsulate hydrophobic carotenoids such as β-cyclodextrin, β-lactoglobulin, and amylose. The interaction of inclusion is shown in Figure 9.

Cyclodextrin is a cyclic oligosaccharide and is classified into different types based on the number of glucose residues, and it is the most well-studied complex ligand involving encapsulated carotenoids. Carotenoids can be encapsulated in the hydrophobic cavity of cyclodextrin through non-covalent interactions to stabilize the carotenoids with a random transformation from a crystalline to an amorphous state [84], and the hydrophilic outer surface of cyclodextrin helps the dissolution of carotenoids. Encapsulation in α-, β-, and γ-cyclodextrin was studied in tomato oil, which contained a lot of carotenoids. The complex presented as the microcrystalline in the emulsion form, and the complex powder was obtained with lyophilization to remove the solvent, accompanied by higher encapsulation and higher antioxidant capability [85]. The β-carotene/β-cyclodextrin complex was proposed, and β-carotene existed as an amorphous state in the complex, as shown in the results of DSC and XRD, leading to a 10- and 40-fold higher solubility and stability, respectively. Moreover, the antitumor activity was also improved [86]. The β-carotene/2-hydroxylproply-β-cyclodextrin/carrageenan/soy protein complex was also proposed and presented as an amorphous state in the DSC thermogram; it showed excellent bioaccessibility (78%) [87]. Astaxanthin has been prepared as a complex as well. The hexatomic side ring of astaxanthin was incorporated into the cavity of methyl-β-cyclodextrin to form a complex in an amorphous state, which was proved by the DSC thermogram, and the product exhibited 54-fold higher solubility, a 10-fold dissolution rate, and better bioaccessibility [88]. Sun et al. [81] proposed fucoxanthin (FX)/2-hydroxylpropyl-β-cyclodextrin via sonication and spray drying. The results of FTIR revealed that the characteristic peaks of FX disappeared, indicating that FX may have been successfully encapsulated, and the molecular docking suggested hydrogen bonding between FX and 2-hydroxylpropyl-β-cyclodextrin. XRD analysis also confirmed the amorphous state of FX. The FX/2-hydroxylpropyl-β-cyclodextrin complex showed better stability and antitumor activities toward HCT-116 and Caco-2 cells compared with free FX.

Amylose is also a food polymer that can accommodate hydrophobic carotenoids via V-amylose crystalline formation, and whether complexes are formed depends on the size, shape, and hydrophobicity of APIs. V-amylose is produced by the addition of ethanol as a precipitant; however, ethanol cannot incorporate into the hydrophobic cavity, and a hydrophobic carotenoid will be trapped by a nonspecific or specific interaction and stabilized with amylose polycrystals [89,90,91] which may be why APIs exist as amorphs in the amylose complex. However, among carotenoids, only β-carotene has been studied in the starch-complex system; the major finding was improved stability.

β-lactoglobulin is also regarded as a complex ligand for carotenoids, existing as the principal protein in whey protein. It can possibly bind hydrophobic carotenoids via the internal cavity of the β-barrel, the surface near Trp^19^-Arg^124^, and the groove between the α-helix and β-barrel of β-lactoglobulin [83,92]; the interaction may lower the molecular mobility to prevent recrystallization [93]. The binding mechanism has been studied in lycopene [94], but the effects on the bioactivity and physicochemical properties caused by the crystalline alteration still need to be further studied.

### 5.4. Micro/Nano Particles

**Figure 10:**
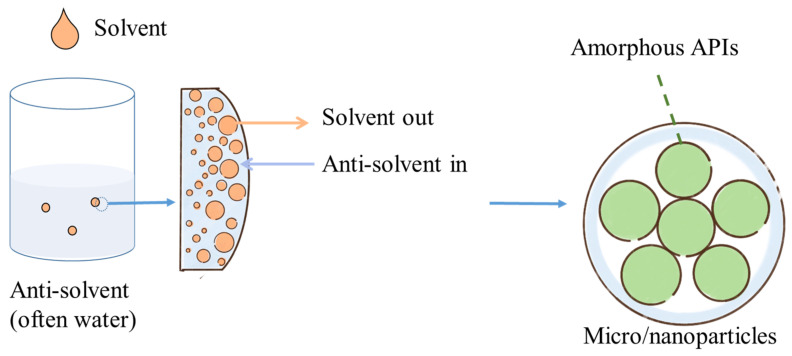
The mechanism of trapping amorphous APIs in nanoparticles via nanoprecipitation [96].

Microparticle preparation includes spray drying, hot-melt extrusion, and phase separation [95], and the removal of solvents in spray drying as well as the cooling of the melt in extrusion causes APIs to remain in an amorphous state. In terms of phase separation, it works in a way similar to that of nanoprecipitation, which is the most common method used for nanoparticle preparation. The main concept of nanoprecipitation is solvent shifting, namely, the ouzo process. The schematic diagram of nanoprecipitation is shown in Figure 10, where hydrophobic APIs and polymers are dissolved in an organic solvent, and the organic solvent is added dropwise into an antisolvent (typically water). The solvent in the droplets moves toward the antisolvent, and the antisolvent does the reverse, causing a supersaturated state. This supersaturated state will further lead to the coprecipitation of hydrophobic APIs and the hydrophobic moiety of polymers forming nanoparticles with the surface coverage of the hydrophilic moiety of the polymers. The precipitated APIs remain amorphous as they do not have enough time to recrystallize in such a quick solvent replacement [96,97].

In our previous study about carotenoid nanoparticles [98], β-carotene-loaded PLGA-PVP nanoparticles were proposed, where amorphous β-carotene may benefit the solubility and oral absorption. Better oral absorption may result from nanoparticle morphology. Hu et al. [99] found that smooth and globular nanoparticles without irregularly lumpy astaxanthin crystals penetrate more easily into the cell. The theory has been applied in the micro-scale dimension, where astaxanthin-loaded hydrophilic microcapsules were obtained by spray drying. XRD analysis indicated that astaxanthin was encapsulated in an amorphous state, and the HepG2 cell growth inhibition activity was boosted [100]. Lutein and PVP have been prepared as particles to increase solubility and stability. PVP can inhibit crystallization by reducing molecular mobility, and the hydrogen bonding with lutein stabilizes the amorphous lutein, accompanied by higher stability against heat, light, and oxygen [101]. Lutein was also incorporated in zein nanoparticles and exhibited 80-fold higher water solubility. No crystalline peaks of lutein were found in XRD analysis as the nanoconfinement restricted the crystallization [102] and resulted in higher cellular uptake. Without excipients, the optical properties of β-carotene, lycopene, astaxanthin, and lutein nanoparticles obtained via nanoprecipitation were compared. With this preparation, the shell of the nanoparticles remained amorphous, and the core was still crystallized, as shown in cryo-TEM images. Moreover, the effective conjugation length of amorphous molecules was shorter than that of bulk crystals, and the absorption wavelength of amorphous molecules was blue-shifted. As a result, the color of the nanoparticles and crystals was rendered yellow and red, respectively [103]. In addition to the amorphous form, a new crystal may appear in the formulation. Ling et al. [104] proposed astaxanthin colloidal particles, and the decreased crystallinity led to a higher dissolution rate. Notably, astaxanthin has two common crystalline forms: polymorph I and polymorph II. A different polymorph was observed within the colloidal nanoparticle in the XRD analysis.

### 5.5. Lipid-Based Formulations

**Figure 11:**
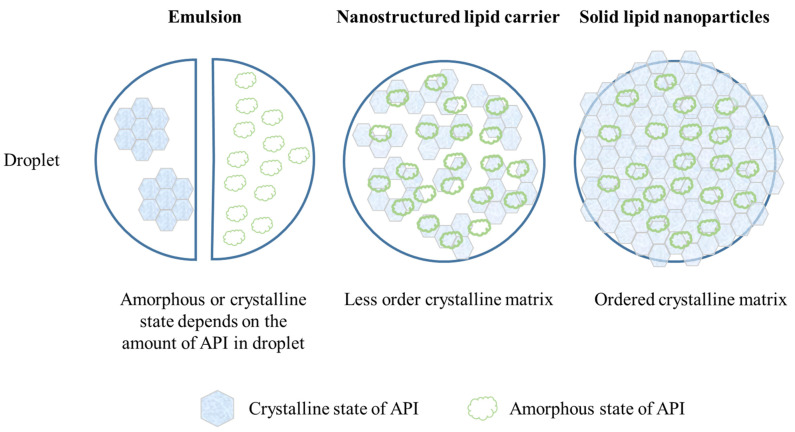
Illustration of a lipid-based formulation.

Lipid-based formulations, including emulsions, solid lipid nanoparticles (SLNs), nanostructured lipid carriers (NLCs), and self-emulsifying drug delivery systems (SEDDSs), are suitable for developing active lipid-soluble compounds, such as carotenoids, for oral bioavailability improvement. Emulsions, SLNs, and NLCs are composed of an aqueous phase and a lipid phase, with a surfactant for stabilization. The lipids used in these formulations are liquid, solid, and a mixture of liquid and solid oil, respectively (Figure 11). SEDDSs contain only oil and surfactants without water. Crystalline APIs would first be dissolved or melted to disperse in the oil to maintain the liquid state during the preparation procedure, so the APIs may be a solution type. Crystallization may occur after homogenization, cooling, or the storage period. In addition, supersaturation also causes the crystalline condition; therefore, the solubility of crystalline APIs in the solvent (solid lipid and liquid lipid) is crucial for crystallization. The general solubility equation (Equation (3)) is always utilized to calculate the solubility of crystalline APIs in a solvent using easily measurable properties [105].

Log S_w_ = 0.5 − 0.01 (T_m_ − 25) − logK_OW_ (3)

where S_W_ is the molar water solubility, T_m_ is the melting point, and K_OW_ is the oil–water partition coefficient of the solute.

**Figure 12:**
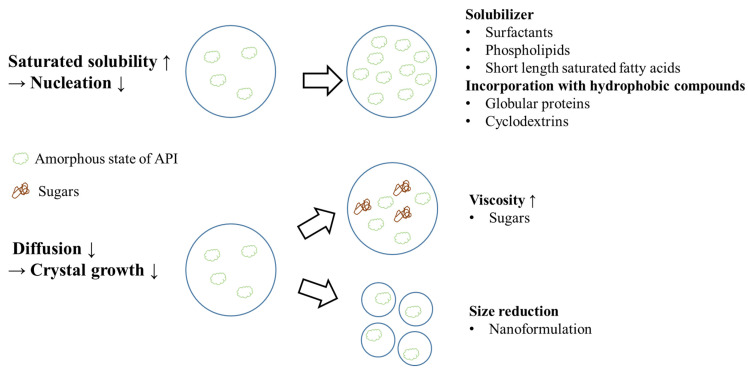
The mechanism of crystallization suppression in a lipid-based formulation.

For crystallization suppression, there are two main strategies: (i) enhancement of the saturated solubility to impede nucleation, and (ii) slowing down the diffusion for the prevention of crystalline growth (Figure 12). For the enhancement of the saturated solubility, the addition of surfactants, phospholipids, or high polarity, short-length saturated fatty acids could form micelles to reduce the driving force of nucleation and prevent crystal formation. Furthermore, the addition of non-polar agents, such as globular proteins or cyclodextrins, on their surfaces for incorporation with hydrophobic active compounds could also enhance the saturated solubility [55,105]. In order to slow down diffusion, viscosity changes and size reductions are often used. In a previous study, the addition of sugars enhanced the viscosity of the continuous phase to block the APIs’ diffusion, and the growth was retarded [105]. A previous study demonstrated that heterogeneous nucleations are confined when the average diameter of the drops is reduced and the crystallization is limited [54].

Apart from these strategies to prevent crystallization, the selection of lipids may affect the crystallization of imperfect crystalline or amorphous states and have different drug loadings, sizes, charges, and release behaviors [106]. The commonly used solid lipids in this formulation preparation contain triglycerides, waxes, fatty acids, and fatty alcohols and the lipid composition may have an influence on the crystalline state of SLNs/SLMs [107]. Taking the commonly used solid lipids, triglycerides as an example, these exhibit polymorphism upon cooling and possibly form the crystalline structure of α, β′, and β crystals with hexagonal, orthorhombic, and triclinic unit structures, respectively. The α-form is the most unstable structure, and spherical particles have been observed when triglycerides are in this form. During storage, the prepared formulation may spontaneously transfer the crystalline structure to a lower-energy state. Some lipids with higher polarity and amphiphilic properties, such as phospholipids, sterols, and di- and mono-acylglycerols, have been regarded as crystallization modifiers and affect the crystallization process [108]. In addition, the type of emulsifier may also affect the crystallization of lipid-based formulations [109]. The longer alkyl chain length of the surfactants has been shown to enhance the crystallinity of lipid-based formulations [107]. Thus, both the lipids and surfactants used in the formulation play a vital role in the modulation of the crystallization process. More research on each formulation is discussed as follows.

#### 5.5.1. Solid Lipid Nanoparticles/Microparticles

The composition of solid lipid nanoparticles/microparticles (SLNs/SLMs) is similar to that of emulsions, but solid lipids are applied in the oil phase to have controlled release behavior or particle stability. The encapsulated drug may be prevented from crystallization and then form a solid solution. The distribution of carotenoids in SLNs may be uniform due to the high hydrophobicity and crystallization temperature [110]. High-energy-state α-crystals were found in the lipids of initial SLNs owing to rapid cooling, and the α-crystals may transfer to β-forms during storage [111]. Compared to α-crystals, highly lipophilic compounds such as carotenoids tend to be expelled under the β-crystalline state and the drug content may be reduced to affect the therapeutic effect. Therefore, the stability of SLNs is usually mentioned as a concern [112].

Some research has been reported about carotenoids containing SLNs/SLMs. β-carotene has been developed as an SLM using stearic acid and sunflower oil to prevent degradation during 7-month storage. The addition of sunflower oil resulted in less ordered crystals and induced an amorphous state, indicating the mixture of long-chained solid lipids and liquid lipids was suitable for the preparation of stable SLMs to prevent β-carotene degradation and exhibit excellent bioactivities [113]. Chen et al. [114] used both palm stearin and cholesterol as the solid lipid carrier of fucoxanthin to avoid the highly ordered crystalline structure of single solid lipids. The results showed that the SLN-microcapsules exist in an amorphous state owing to anti-solvent precipitation and ultrasonic treatment to form micelles easily and are capable of absorption by intestinal epithelial cells, indicating that the solubility of carotenoids could be enhanced to reduce the driving force of nucleation. The higher glass transition temperature indicated that the formulation has better temperature resistance [115]. Zeaxanthin was also prepared for SLNs using glycerol monostearate or glycerol distearate to resolve the problem of lipophilicity and instability. The crystallinity was determined to decrease in the formulation via the examination of DSC with the melting enthalpy decrease manner. The crystals of lipids preferred to form α-crystals with the high-energy state during the rapid cooling procedure [116]. Glycerol distearate is a mixture of C16 and C18 fatty acids and has a relatively low melting point and enthalpy compared to glycerol monostearate, indicating it has a poor crystalline structure. Therefore, SLNs prepared using glycerol distearate have the irregularity of the lipid crystals and display greater dissolution behavior [116].

#### 5.5.2. Nanostructured Lipid Carrier

Nanostructured lipid carriers (NLCs) are similar to nanoemulsions and SLNs, but the lipids in NLCs include not only solid lipids but also liquid lipids. The incorporation of liquid lipids can allow the internal lipid phase to have a less-ordered crystalline arrangement to obviate the condition of active compound leakage and load more active compounds. Owing to the composition of both solid and liquid lipids, crystallinity is always considered. The crystalline index (CI) was reported to determine the crystalline state of APIs loaded in NLCs and it is calculated using Equation (4). A higher CI indicates that the encapsulation efficiency of NLCs may be higher owing to the less-ordered crystalline arrangement [106].

(4) $$\mathrm{CI}\;(\%)=\frac{M_{s}}{M_{p}\;\times \;\gamma }\;\times \;100$$

where M_s_ indicates the melting enthalpy of NLCs, M_p_ indicates the melting enthalpy of pure solid lipids, and γ indicates the solid lipid concentration (%) in NLCs.

Astaxanthin-loaded NLCs have been developed to improve the physicochemical characteristics and storage stability. Glyceryl behenate and oleic acid were selected as solid and liquid lipids and lecithin and Tween 80 were chosen as surfactants in the oil phase and water phase, respectively. The authors evaluated the properties of NLCs prepared via lecithin removal, replacing Tween 80 with Tween 20 or replacing oleic acid with triacylglycerols, and the results showed that there is no improvement in the stability of the NLCs due to the chemically homogenous structure of the lipid mixture. In these formulations, β-crystals were formed and the aggregation, which contributed to the hydrophobic interaction, partial coalescence, or the penetration of lipid crystals, made the NLCs unstable. Among these solid lipids, NLCs prepared using glyceryl behenate were reported to have a more imperfect crystalline lattice and lead to high stability and entrapment. The crystal was demonstrated to be a metastable β′ polymorph, and the reduction in crystallinity compared to glyceryl behenate was determined by the broader and lower intensities in the XRD patterns and DSC thermogram. The incorporation of bioactive compounds may also make more imperfect crystals, leading to better encapsulation efficiency. The melting point of astaxanthin disappeared in the NLC group, indicating that astaxanthin is not in the crystalline state at this temperature and can be considered to be physically stable at high temperatures [115]. Oleic acid has been reported as a crystallization inhibitor. Oleic acid can adsorb and crystallize at the surface in the beginning so the crystallization may be hindered [54].

Glycerol monostearate or glycerol distearate as a solid lipid, medium-chain triglycerides as a liquid lipid, and soy lecithin and Tween 80 as surfactants were used to prepare NLCs to load zeaxanthin. The enthalpy reduction was observed in the NLC group compared to SLNs, indicating the crystallinity decrease in the lipid matrix. The lower enthalpy and crystallinity are capable of encapsulating more APIs and displaying better release behavior. Similar to the results of SLNs, NLCs prepared using glycerol distearate also had better properties. The results showed that the incorporation of liquid lipids and the selection of lipids have an influence on the crystallinity, and further improve the physicochemical characteristics of active compounds and formulations [116].

#### 5.5.3. Microemulsion/Nanoemulsion

An emulsion is a mixture of two immiscible phases (aqueous phase and liquid oil phase) supported by a surfactant to reduce surface tension under thermodynamically unstable conditions. Solubilized and crystallized β-carotene nanoemulsions have been prepared to compare the influence of physical properties on bioaccessibility. The crystalline state was examined while passing through the stimulated digestion process, and no crystal was observed during digestion in the solubilized β-carotene nanoemulsion group. In the crystallized β-carotene nanoemulsion group, the initial crystals gradually disappeared and possibly contributed to the dilution by digestive juice in each step. The free fatty acid release profiles in the in vitro digestion study indicated that the physical state of β-carotene has no influence on lipid digestion. The bioaccessibility results showed that the solubilized β-carotene nanoemulsions had an 11.7- and 46-fold enhancement compared to crystallized β-carotene nanoemulsions and crystallized β-carotene in phosphate buffer saline, respectively, suggesting that the solubilization state without crystals is the suitable delivery strategy [117]. A previous study developed a lutein-loaded whey protein emulsion, which is similar to a Pickering emulsion. The crystalline form of the formulation containing only whey protein and phospholipids could be observed using a microscope, and the situation could be improved after the addition of mono- and di-glycerides. The mono- and di-glycerides benefited the solubility of lutein crystals and were demonstrated to be physical barriers in the crystalline growth process to prevent the carotenoids from crystallization, as well as improve the stability [118].

#### 5.5.4. Self-Emulsifying Drug Delivery System

**Figure 13:**
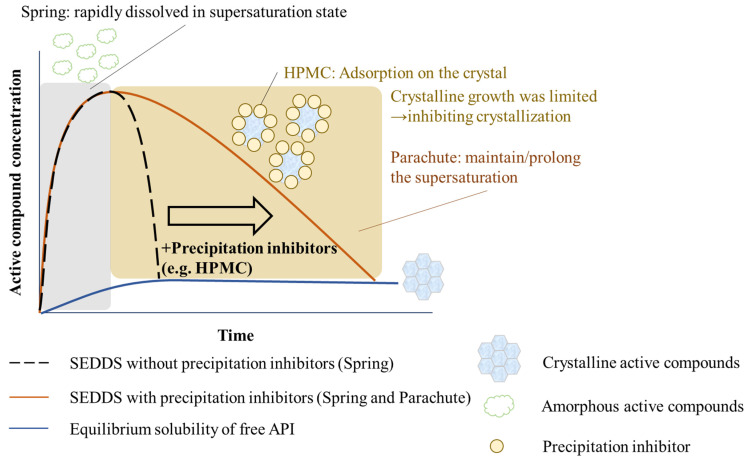
The mechanism of the “Spring and Parachute”.

A self-emulsifying drug delivery system (SEDDS) is a mixture of active compounds, oils, surfactants, and co-surfactants through a gentle stirring procedure and the o/w emulsion is obtained by contact with digestion fluids and digestive motility. SEDDSs can be divided into two groups according to the droplet size. The droplet size of self-microemulsifying drug delivery systems and self-nanoemulsifying drug delivery systems is 100–250 nm and less than 100 nm, respectively [119]. A SEDDS is often in an amorphous state owing to being dissolved in lipids and surfactants. The crystallization always occurs during digestion due to the supersaturation to make excess active compounds precipitate or crystallize. In order to prevent supersaturation, some precipitation inhibitors have been reported, including cellulose (HPMC and hydroxypropyl cellulose), polymers (PVP and Soluplus^®^), surfactants (Tween, Cremophor, and D-α-Tocopherol polyethylene glycol 1000 succinate), and cyclodextrins [120,121]. The mechanism of the commonly used precipitation inhibitor HPMC, is to adsorb onto the surface to inhibit the nucleation and growth and to form intramolecular and intermolecular hydrogen bonds between the active compounds and itself via the hydroxyl groups in the structure. In addition, the “Spring and Parachute” is also observed in formulations with these excipients. Supersaturation may be rapidly reached, displaying spring behavior and the nucleation or crystalline growth may be restrained to stabilize the metastable supersaturated samples, witnessing the parachute phenomenon (Figure 13). In this way, the precipitation is inhibited, and there is a longer time for absorption in the expected regions [121]. The degree of supersaturation (S) is driven to precipitate, and it can be calculated using Equation (5) [122].

(5) $$S=\frac{\;\mathrm{total}\;\mathrm{drug}\;\mathrm{concentration}}{\mathrm{saturation}\;\mathrm{concentration}\;\mathrm{of}\;\mathrm{the}\;\mathrm{drug}\;\mathrm{in}\;\mathrm{the}\;\mathrm{solvent}}$$

A SEDDS was incorporated with a solid dispersion, named a lipid-based solid dispersion (LBSD), to load lycopene to enhance the dissolution characteristics and oral absorption of lycopene. The results of XRD and DSC indicated that new signals appeared in the LBSD and that it had a lower melting point. The release behavior of the amorphous marketed product, Lycovit^®^, was significantly increased compared to the lycopene crystals, suggesting the benefit of crystalline transition. Although no obvious enhancement was observed in the LBSD owing to the non-amorphous state, the pharmacokinetic study demonstrated a significant improvement in oral absorption compared to Lycovit^®^ due to the long-chained triglyceride for lymphatic transportation, suggesting the crystalline state did not fully affect the oral absorption in this study [123]. Aung et al. [124] prepared astaxanthin-loaded SMEDDS tablets containing SMEDDS, hydrophilic polymers as precipitation inhibitors, and microcrystalline cellulose for tableting. The SMEDDS, composed of rice bran oil, Kolliphor^®^ RH 40, Span^®^ 20, and two polymers, HPMC and polyvinyl alcohol, was used to obtain the supersaturation state and enhance the release of astaxanthin. The crystallinity was determined by PXRD and transferred from a crystalline to amorphous state after preparing the supersaturable SMEDDS. The SMEDDS with or without precipitation inhibitors enhanced the release behavior, antioxidant activity, and cellular uptake. The precipitation inhibitors in the SMEDDS could hinder the nucleation and precipitation and thus maintain astaxanthin in the solubilized form.

### 5.6. Liposome

**Figure 14:**
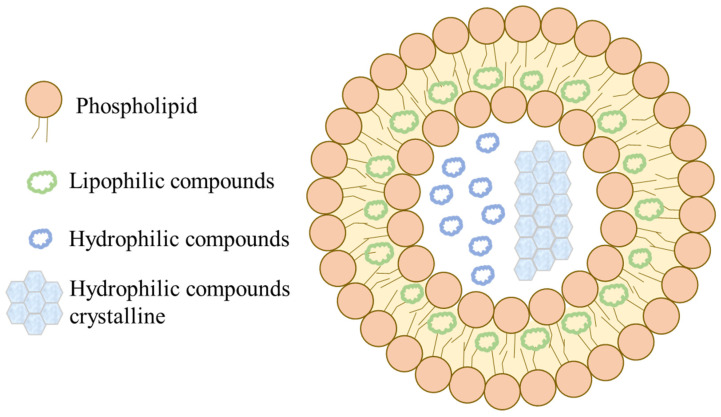
Schematic of the hydrophilic and lipophilic compounds within a liposome. The right indicates that the hydrophilic compounds in the core may undergo crystallization and precipitation.

The liposome is composed of a hydrophobic phospholipid bilayer shell and a hydrophilic core to form a spherical structure. Active lipophilic compounds tend to be encapsulated within the lipid bilayer and not precipitate in a crystalline form. Regarding the active hydrophilic compounds, they were loaded in the aqueous core with the crystalline precipitate, amorphous precipitate, or solution state depending on the properties of the active compounds and preparation process (Figure 14) [125]. Carotenoids are lipophilic compounds with high octanol–water partition coefficients; therefore, they are usually encapsulated within the phospholipid bilayer. Astaxanthin has been prepared for the liposome using the film dispersion-ultrasonic technique. XRD was conducted to examine the crystalline state of astaxanthin, soybean phosphatidylcholine (excipient), and the liposome. Pure astaxanthin was determined to be in a crystalline state owing to the existence of many peaks. The pattern of liposomes was different from that of astaxanthin and soybean phosphatidylcholine, implying that astaxanthin was successfully encapsulated in the liposomes with hydrogen bonds between astaxanthin and the phospholipid bilayer. The aqueous solubility of astaxanthin was observed to be enhanced 17-fold compared to pure astaxanthin, which may be attributed to the crystalline alternation of astaxanthin after liposome encapsulation [126].

Although there are some studies illustrating other factors majorly affecting oral absorption, such as intestine-specific transporters or lymphatic transport, solubility enhancement was mainly discussed and proved to efficiently improve oral bioavailability. The simplest way to enhance solubility is to alter the crystalline state from crystals to amorphs or polymorphs. The limitation is the instability of crystals owing to the high-energy state. To overcome this problem, there are many strategies and principles for crystallization suppression discussed in this article. To summarize, carotenoid formulations involved in the change of crystallinity are listed in Table 2. This article included various carotenoid-containing formulations, including those prepared using polymers or lipids, and the discussion of crystalline alternation. It may provide information to develop carotenoid-loaded formulations to deal with the problem of solubility and stability and exhibit outstanding bioactivities.

## 6. Conclusions

This review deals with the mechanisms of converting crystals into amorphs and stabilizing the amorphs in terms of polymer- and lipid-based formulations. Factors such as the types of excipients, manufacturing processes, and changes in particle size can transform the crystalline forms of APIs into other polymorph or amorph statuses. This review also provides representative and practical strategies for the delivery of carotenoids. These pharmaceutical technologies related to crystalline status modification efficiently improve the physicochemical properties of carotenoids, which amends their oral bioavailability and biological effects.

## Figures and Tables

**Table 2:** The summary of carotenoid formulations involved in the crystalline change.

Carotenoids	Formulation	Composition	Crystalline Status	Results	Reference
Carotenoids	Co-crystal	Sucrose	Crystals	Thermal stability↑	[70]
β-carotene	Solid dispersion	Poly (vinyl pyrrolidone) Sucrose fatty acid ester (S-1670)	Amorphs	Solubility↑ Dissolution↑ Bioavailability↑	[75,76,77]
β-carotene	Solid dispersion	Cyclic amylopection	Amorphs	Stability↑	[78]
β-carotγene	Inclusion complex	Amylose (Amylomaize starch)	Both Amorphs and crystals	Stability↑	[89]
β-carotene	Inclusion complex	Amylose (Corn starch)	Both Amorphs and crystals	Stability↑	[91]
β-carotene	Inclusion complex	Amylose (High-amylose corn starch)	Amorphs	Stability↑	[90]
β-carotene	Inclusion complex	2-hydroxylproply-β-cyclodextrin Carrageenan Soy protein	Amorphs	Bioaccessibility↑	[87]
β-carotene	Nanoparticles	Poly (lactic-co-glycolic) acid Poly (vinyl pyrrolidone)	Amorphs	Bioavailability↑	[98]
β-carotene	Nanoemulsion	Corn oil	Amorphs	Bioaccessibility↑	[117]
β-carotene	Solid lipid microparticles	Stearic acid Sunflower oil	Amorphs or Less ordered crystals	Stability↑	[113]
Lycopene	Solid dispersion (Dripping pills)	PEG 6000 Cremophor^®^ EL Tween^®^ 80	Amorphs	Dissolution↑ Bioavailability↑	[79]
Lycopene (Tomato oil)	Inclusion complex	α, β, γ-cyclodextrin	Microcrystals	Color change Stability↑ Antioxidation↑	[85]
Lycopene	Lipid based solid dispersion	Gelucire 44/14	Polymorphs	Dissolution↑ Bioavailability↑	[123]
Astaxanthin	Inclusion complex	Methyl-β-cyclodextrin	Amorphs	Solubility↑ Dissolution↑ Bioaccessibility↑	[88]
Astaxanthin	Colloidal particles	Tween^®^ 20 Sodium caseinate Gum arabic	Polymorphs	Dissolution↑ Cellular uptake↑	[104]
Astaxanthin	Microparticles	Povidone K30 Copovidone PEG 6000 Poloxamer 188 Tocopherol Colloidal silicon dioxide	Amorphs	HepG2 cell growth inhibition activity↑	[100]
Astaxanthin	Nanoparticles	Poly(lactic-co-glycolic acid)	Amorphs	Cellular uptake↑ Photoprotection↑	[99]
Astaxanthin	Liposome	Soybean phosphatidyl choline Cholesterol	Both Amorphs and crystals	Solubility↑ Stability↑	[126]
Astaxanthin	Nanostructured lipid carrier	Glyceryl behenate Oleic acid Lecithin Tween^®^ 80	Amorphs	Stability↑	[115]
Astaxanthin	Self-microemulsifying drug delivery system	Rice bran oil Kolliphor^®^ RH 40 Span^®^ 20 HPMC Polyvinyl alcohol	Amorphs	Dissolution↑ Antioxidation↑ Cellular uptake↑	[124]
Fucoxanthin	Inclusion complex	2-hydroxylpropyl-β-cyclodextrin	Amorphs	Solubility↑ Stability↑ Anti-tumor activity↑	[81]
Fucoxanthin	Solid lipid nanoparticle-microcapsules	Palm stearin Cholesterol	Amorphs	Solubility↑ Stability↑ Bioavailability↑	[114]
Lutein	Particles	Polyvinylpyrrolidone Tween^®^ 80	Amorphs	Stability↑	[101]
Lutein	Nanoparticles	Zein Sophorolipid	Amorphs	Solubility↑ Bioaccessibility↑	[102]
Lutein	Nanoemulsion	Blending plant oil Mono- and di-glycerides Lecithin Whey protein	Amorphs	Dissolution↑ Stability↑	[118]
Zeaxanthin	Solid lipid nanoparticles	Glycerol monostearate Glycerol distearate	Possible amorphs	Dissolution↑	[116]
Zeaxanthin	Nanostructured lipid carrier	Glycerol monostearate Glycerol distearate Medium-chain triglyceride Soy lecithin Tween^®^ 80	Possible amorphs	Dissolution↑	[116]

↑: enhancement. ↓: reduction.

## Data Availability

Not applicable.
